# Decision Tree-Based Body Constitution Diagnosis System for Traditional Chinese Medicine

**DOI:** 10.1155/2022/5560087

**Published:** 2022-03-07

**Authors:** Cheng-Chan Yang, Shi-Jim Yen, Xian-Dong Chiu, Kuo-Chu Wu, Shih-Cheng Ye, San-Hua Su, Hsiao-Yi Huang

**Affiliations:** ^1^Department of Chinese Medicine, Hualien Tzu Chi Hospital, Buddhist Tzu Chi Medical Foundation, Hualien, Taiwan; ^2^School of Post Baccalaureate Chinese Medicine, Tzu Chi University, Hualien, Taiwan; ^3^Department of Computer Science and Information Engineering, National Dong Hwa University, Hualien, Taiwan

## Abstract

This study aimed to establish a method for fast and accurate determination of body constitution types from the body constitution questionnaire (BCQ) by employing a decision tree model. The model was trained for 4 classes, namely, Yin-Xu, Yang-Xu, Phlegm and Blood Stasis, and Normal, and it achieved 67% accuracy for the testing dataset. The model also reduced the required number of BCQ questions from 44 to 3–6, depending on the responses. Lastly, we developed the Traditional Chinese Medicine (TCM) body constitution online diagnosis system using our model to collect data digitally and use it more practically and efficiently. This system can assist doctors to improve the diagnosis and treatment in TCM practice.

## 1. Introduction

In Traditional Chinese Medicine (TCM) practice, body constitution (BC) is the core theoretical basis for determining an individual's health status. The BC type affects individuals' susceptibility to specific diseases and strongly influences their prognoses. Therefore, being able to quickly and accurately determine a patient's BC is an important issue in TCM clinical practice. A body constitution questionnaire (BCQ) is an objective tool that can help bridge the gap between TCM's individualized medical features and scientific research methodology. Syndrome differentiation is one of the most important concepts in the diagnostic process of TCM. Decision tree algorithms are appropriate for this process as they can address a large amount of variable information to obtain more precise and accurate classifications. The algorithms can also handle incomplete data. Therefore, decision tree algorithms can help clinicians determine the relationship between symptoms/signs and syndromes, thereby improving the syndrome differentiation and treatment process in TCM.

In this study, we used the decision tree algorithm to optimize the process of using the TCM BCQ to determine patients' BC type. The study background and previous related research, materials and methods, results, discussion, conclusion, and future research directions have been presented herein.

### 1.1. Background and Previous Research

The four cardinal TCM diagnostic methods (seeing, smelling, asking, and touching) depend on the physician's subjective observations, knowledge, and clinical experience [1]. Moreover, TCM traditionally relies on subjective information—including the physician's perception and the patient's chief complaint—to reach a clinical diagnosis [2, 3]. Therefore, the objectivity of diagnosis in TCM practice and its scientific basis have often been contested [4]. To address this issue, several efforts have been made to improve TCM diagnosis methods. BC is the fundamental theory of TCM [5]. BC indicates the individuals' physiological characteristics: their susceptibility to pathogenic factors and tendency to develop certain types of pathological changes [6]. Because the BC type determines an individual's susceptibility to specific diseases and has prognostic relevance, it is used to guide treatment and disease-prevention measures [7]. Different BC types also have specific metabolic characteristics. Based on an individuals' BC, TCM practitioners advise personalized preventive and therapeutic measures, thereby achieving better treatment outcomes [5]. Currently, two Chinese Medical Constitution Questionnaire tools are widely used to determine the BC type: The Constitution in Chinese Medicine Questionnaire (CCMQ) [8] and BCQ [9, 10]. Lin [11] assessed the differences between the two questionnaires. Although clinical studies using the BCQ [9, 10] have reported promising results in recent years, these studies only evaluated and compared the questionnaires without addressing their ease of use.

The BCQ consists of 44 questions, each with a maximum score of 5 (ranging from 1 (never happened) to 5 (always happen)); the total score (calculated by summing the scores of all items) ranges from 44 to 220. The questions are aimed at determining the BC type, classified into 3 categories: Yang-Xu (19 questions), Yin-Xu (19 questions), and Stasis (16 questions). Some questions are used to determine more than one type. For Yang-Xu, a score exceeding 31 implies Yang-Xu BC; for Yin-Xu, a score exceeding 30 implies Yin-Xu BC; and for Stasis, a score exceeding 27 implies Stasis BC. The higher the score, the more obvious is the tendency to represent the BC. Scores less than the threshold for all three BC types are considered indicative of a peaceful constitution [9]. Previous studies have demonstrated satisfactory reliability (Cronbach's *α*: 0.85–0.92) and validity (*z* score: 3.3636–10.026) of the BCQ [12]. In recent years, the BCQ has been increasingly used in clinical research on several diseases, such as schizophrenia [13], breast cancer [14, 15], and diabetes [16, 17], and the assessment of the Yang-Xu constitution and clinical blood variables [18]. Although the 44 questions in the BCQ were used in these studies, the research did not attempt to make the process of using this tool simpler and more convenient.

In recent years, the concept of “big data” has been applied to assess the relationship between the intervention measures and the outcomes of diseases. The advent of big data technology provides great opportunities for the modernization of TCM [19]. A decision tree is a kind of inductive reasoning algorithm that uses the decision tree predictive model to show how the data are affected by various variables; in addition, it uses the dendrogram for automated data segmentation and evaluation [20]. Syndrome differentiation is one of the most important concepts in TCM practice, which is based on a series of diagnostic procedures. The process of syndrome differentiation entails an analysis of the symptoms and signs of the disease at the pathological stage. The syndrome information is complex and diverse and largely consists of qualitative variables. The decision tree can help process information with large amounts of variables and achieve more precise syndrome classification; moreover, it can handle incomplete data. Therefore, the decision tree technology can help determine the relationship of symptoms and signs with syndromes and improve the process of syndrome differentiation and treatment in TCM [21]. Many studies have demonstrated the applicability of decision trees to explain the rules of TCM diagnosis systems based on large TCM syndrome datasets [22]. However, these decision trees may produce huge branch systems, requiring further pruning of the excess branches to increase their efficiency.

The computational origins of decision trees, sometimes called classification trees or regression trees, are models of biological and cognitive processes. These are simple yet effective for predicting and explaining the relationship between some measurements of a variable and their target value. Quinlan developed Iterative Dichotomiser 3 (ID3) [23], C4.5, and C5.0 [24] algorithms. Those decision tree algorithms have helped improve the process of predicting variables and pruning technology.

Liu and Liu [25] used decision trees in the field of medicine, introducing several novel techniques and providing new research directions. Chen et al. [26] and Wang et al. [27] used the decision tree C5.0 module as the basis to construct a diagnostic model to analyze the complex characteristics of chronic hepatitis B in TCM. The decision tree is a tool not only for data analysis but also for extracting clearer judgment rules for physicians as a reference for clinical diagnosis. Although the decision tree theory cited was used in clinical settings, those researchers did not implement a real, operational system using the theory. In contrast, this study developed a mobile device system based on the decision tree theory to validate this theory.

The decision branch is illustrated as a figure much like the branch of a tree. Each node in the tree structure represents a conditional test for an attribute and each branch represents the result of the test; the offspring of the tree represents its final branch. The decision tree is also a type of establishment classification mode, which uses the existing data to produce a tree structure [23, 24, 28]. A tree structure is built by classifying a known instance (i.e., training paradigms), from which hidden rules between fields are summarized. The resulting decision tree can also be used to predict samples.

**Figure 1:**
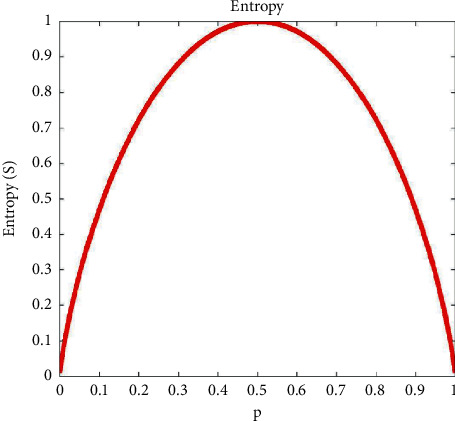
Decision tree entropy.

The decision tree is constructed from the root node from top to bottom and divides the data into subsets containing similar values. In Figure 1, ID3 uses entropy to calculate the uniformity of the sample. If the sample is completely uniform, its entropy is zero; if the sample is equally divided, its entropy is 1 [29].

Entropy using the frequency table of one attribute is expressed as equation (1), where *p*_*i*_ is the probability of class *i* appearing in a dataset *S* with *c* classes.

(1) $$\begin{array}{c} \text{Entropy}\left(S\right)=\overset{c}{\underset{i=1}{\sum }}\left(-p_{i}\log_{2}p_{i}\right). \end{array}$$

Classification and regression trees (CART) are decision tree algorithms that use the Gini index [30]. Gini impurity (calculated using equation (2)), like entropy, is a criterion for splitting nodes in decision trees. The methodology entails the calculation of the “impurity” or “information level” indicator. The node in the decision tree is split according to the information existing on the node using the following formula:

(2) $$\begin{array}{c} \text{Gini}\left(S\right)=1-\overset{c}{\underset{i=1}{\sum }}p_{i}^{2}. \end{array}$$

**Figure 2:**
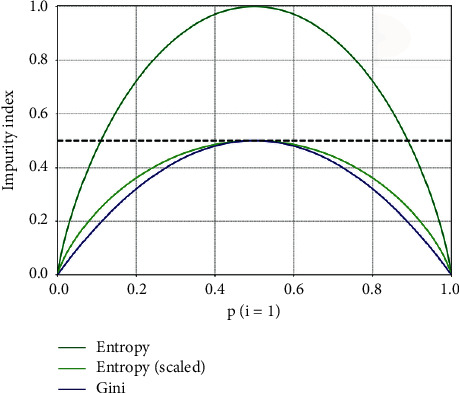
Gini coefficient and entropy.

Different impurity measures (Gini coefficient and entropy) usually produce similar results.  Figure 2 shows that the Gini coefficient and entropy are very similar impurity standards. One of the reasons why Gini is the default value of scikit-learn (Python library) is that the calculation of entropy may be slightly slower (because it uses logarithms).

In the process of optimizing the decision tree, the branches and leaves are pruned to simplify or compress the classification of the unnecessary and redundant parts. Pruning is also a method of compression, which selectively deletes insensitive noncritical and redundant connections in the model, such as noncritical weights or smaller absolute weights [31].

Post-pruning is the most commonly used method for simplifying trees. Because leaves replace the nodes and subtrees, the complexity can increase. Pruning can significantly reduce the size as well as increase the accuracy of the classification. Although pruning is likely to reduce the accuracy of the allocation on part of the test set, the accuracy of the overall tree classification attributes increases [32–34]. Research on pruning methods requires more practical examples to confirm their efficacy. This study applies the pruning method to TCM to obtain a large amount of data that can be used to verify this method's accuracy.

## 2. Materials and Methods

### 2.1. Study Design and Subjects

This study was approved by the Institutional Review Board of Hualien Tzu Chi (IRB number: IRB107-08-B). A total of 439 healthy, mostly young volunteers were recruited from the local community via advertisements between March 2018 and June 2019. The inclusion criteria were as follows: age 20–65 years, no significant medical history, and no current use of medications for chronic illnesses. Written informed consent was obtained from all participants prior to their enrolment. All participants completed the BCQ. Some of the BCQ results represented mixed BC syndrome type. However, we only considered the single BC syndrome type in this study. Consequently, 168 pieces of the BCQ data were selected for the decision tree analysis.

### 2.2. BCQ

The TCM BCQ developed by the research team led by Prof. Yi-Chang Su of China Medical University was used in the study. The questionnaire contains 44 questions. People can understand their BCs by answering whether they are cold, tired, or thirsty.  Table 1 shows the question numbers for each BC.

There are five response options for each question (1, not at all; 2, a little bit; 3, moderate level; 4, very high level, and 5, most serious level). The scores of individual subjects were summed to determine the BC type. For example, there are 19 questions for Yin-Xu, and subjects with >30 points were classified as having Yin-Xu. In this study, subjects with complex constitutions were excluded and only those with a single BC type were included.  Table 2 shows the criteria for determining the BC type.

### 2.3. Decision Tree Analysis

**Figure 3:**
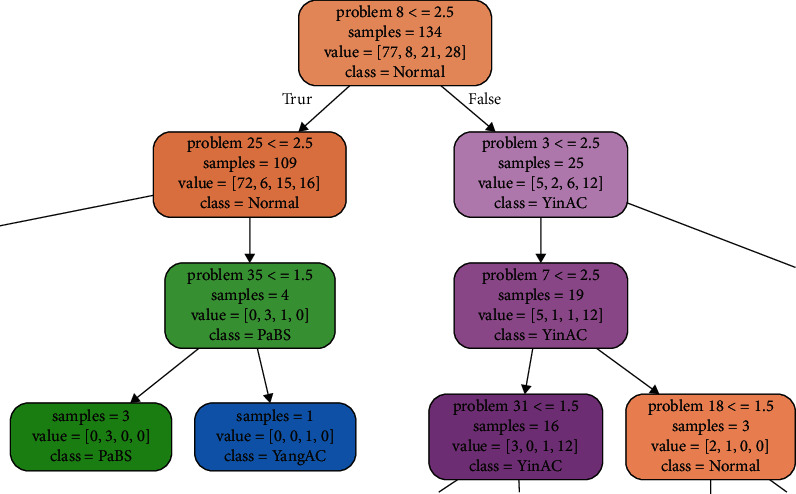
Sample decision tree section.

In total, there were 168 pieces of the BCQ data. In this study, 134 pieces of data were used for training the decision tree, whereas 34 pieces of data were used for testing the decision tree. The decision tree was a CART tree that used the Gini index.  Figure 3 shows one section of the decision tree. Each box is a node representing the result of an answer. Nodes on the left (under the word true) indicate that the judgment condition was satisfied. Nodes on the right (under the word false) indicate that the judgment condition was been satisfied. When there were no items on the left or right under a node, that node indicates the final judgment result. Each node has a specific background color, with brown indicating a normal constitution (normal), green indicating Phlegm and Blood Stasis (PaBS), purple indicating Yin-Xu (YinAC), and blue indicating Yang-Xu (YangAC). The number after the word problem in the first row of each node represents the question number. The value after the greater than (>), less than (<), and/or = symbols represents the answer option (1, not at all; 2, a little bit; 3, moderate level; 4, very high level, and 5, most serious level).

In short, each node on the decision tree asks whether the given question (or condition) has been answered. Those meeting the condition proceed to the node below and left and those not meeting the condition proceed to the node below and right. Depending on the result, it follows the right or left path. The number after sample indicates the number of samples available at that point, and the list of numbers after value indicates how many samples belong to each option category at a given node. The category with the largest number of samples is the predicted value for that node, with a class representing the BC predicted by the node.

As an example, for the root node on the top of Figure 3, the problem number is 8. If the answer is ≤2.5 (true), one should proceed to the box below and to the left. If the answer is ≥3 (false), one should proceed to the node below and to the right. The line sample = 134 means this question has 134 samples. The line value = [77, 8, 21, 28] means the answers are 77 Normal, 8 PaBS, 21 YinAC, and 28 YangAC, respectively. The line class = normal means that the node predicts a BC of normal deficiency.

**Figure 4:**
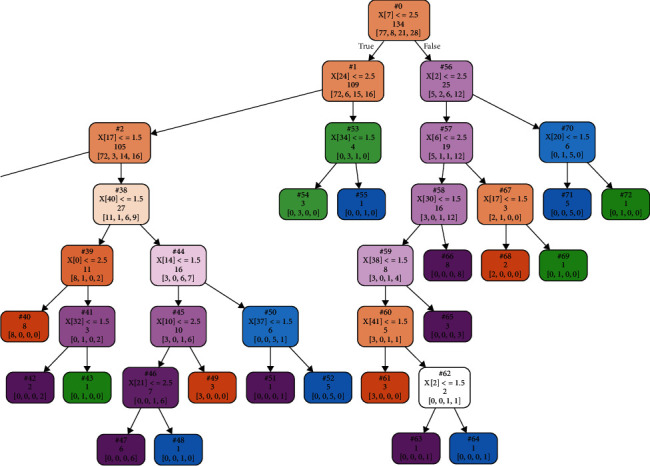
Right half of the entire decision tree.

**Figure 5:**
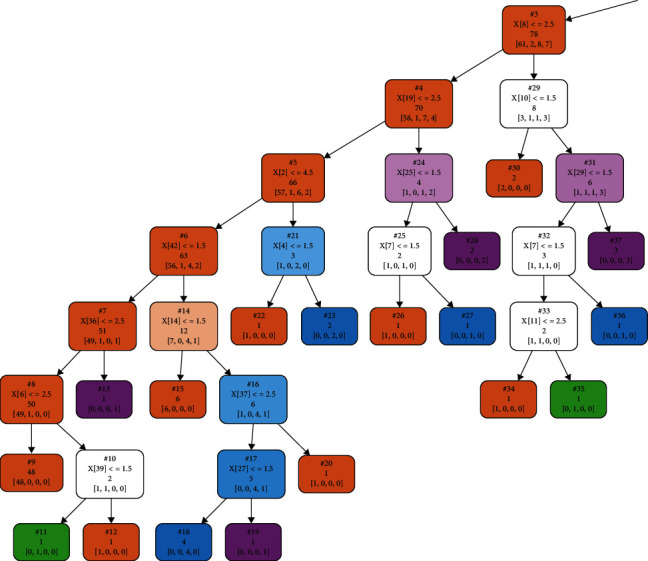
Left half of the entire decision tree.

**Figure 6:**
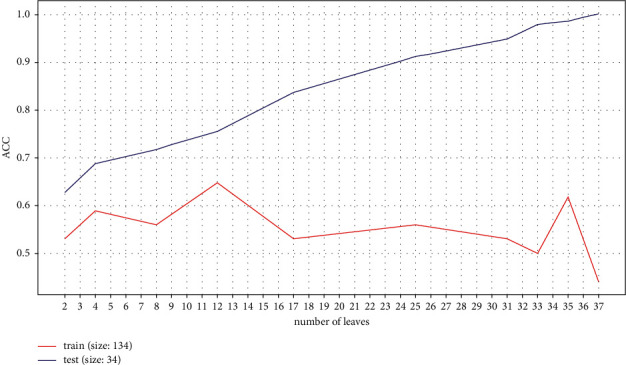
Accuracy of tree size.

Figures 4 and 5 show the entire decision tree. The information in each node is simplified. To optimize this decision tree, we pruned the branches and reduced the redundant parts to obtain the highest accuracy of judgment. We performed an experiment and attempted to use the maximum depth of the decision tree as an independent variable to judge the optimal depth of the decision tree for this study. The experimental result shows that the best accuracy, i.e., 0.67, was obtained with 12 leaves (Figure 6).

In the standard artificial intelligence (AI) system, a larger amount of data are used as a training dataset to establish a clearer and reliable model to verify the reliability and deviation of the system. Subsequently, a small test dataset is used to test the AI system from the perspective of the end-user and to check the accuracy of the results. We adopted the same approach in this study.

**Figure 7:**
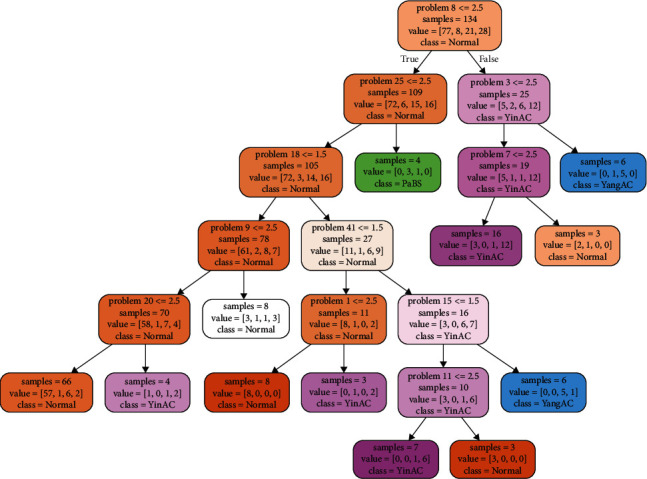
Decision tree after branches were pruned.

Based on the experiments, we found that the optimized decision tree can be reduced to 12 leaves after pruning the branches. As shown in Figure 7, this decision tree was greatly reduced in size without losing its accuracy. This optimization can help reduce the BCQ problems from a maximum of 10 problems to 6 problems for constructing the decision tree.

### 2.4. TCM BC Online Diagnosis System

**Figure 8:**
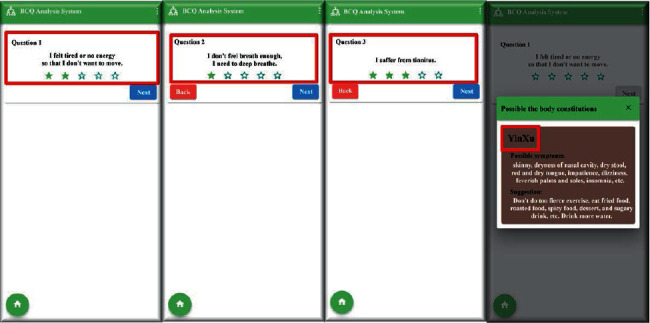
Screenshot of the BCQ app on a mobile device.

The diagnosis system has two parts: the training system and the implementation system. The training system was used to build a decision tree and generate a database of more than 439 subjects for an online application (app). The implementation system was a BCQ app for mobile devices (Figure 8).

**Figure 9:**
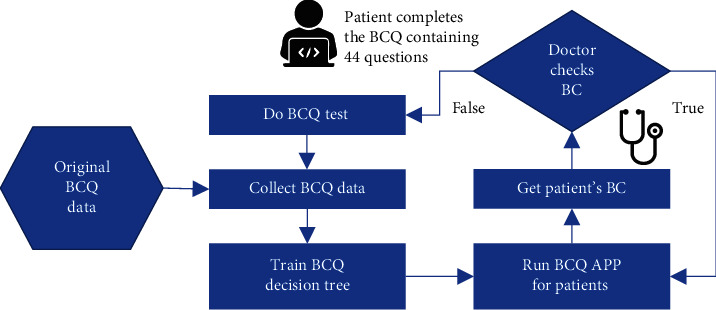
Architecture of the TCM BC online diagnosis system.

The TCM BC online diagnosis system architecture is presented in Figure 9. The system first trains the original BCQ data to the BCQ decision tree. Subsequently, the system displays the questions and options on the BCQ APP. Users can answer questions according to their own circumstances by clicking on the correct choice, as shown in Figure 8. The app then determines the next node based on the response. When a final leaf is reached, the system clearly shows the final judgment of the BC. The doctor may check the judgment in addition to the results of other medical tests to reach a diagnosis for the patient. If the doctor finds the determined BC to be wrong, the patient can be asked to complete the complete BCQ containing 44 questions. The obtained result can then be added to the BCQ data and the BCQ decision tree can be retrained. This may consequently increase the accuracy of the decision tree.

## 3. Results

A total of 439 healthy subjects volunteered to participate in this study. All subjects completed the BCQ without any missing values. Among these, 168 were found to have a single BC syndrome (95 with normal constitution, 25 with Yang-Xu, 36 with Yang-Xu, and 12 with PaBS), whereas 271 were found to have a mixed BC syndrome type. Therefore, we used the dataset of those with single BC and divided it into 2 parts: 134 subjects for training and 34 subjects for testing. We trained our decision tree model using the training dataset. Subsequently, we tested our model using the testing dataset. The prediction accuracy of our decision tree model for the testing dataset was 67%. In addition, we pruned the leaves of the decision tree during training to reduce the size and depth, which allowed us to reduce the number of questions from the original 44 BCQ questions to 3–6 questions for determining the BC type.

Furthermore, we developed a mobile phone app to change the interface for responding to the questionnaire from paper and pen to a mobile device. This innovation simplifies the entire process and allows us to collect more relevant data for future work. The BCQ app is not only helpful in the medical settings but also allows patients to conveniently check their fitness anytime and anywhere.

## 4. Discussion

To the best of our knowledge, this study is the first to apply a decision tree model to the TCM BC concept. As of now, our decision tree model can predict Yin-Xu, Yang-Xu, PaBS, and normal. The prediction result of our model can be used to improve diagnosis and treatment in TCM practice as well as prevent diseases. To accurately assess the BC type, patients need to answer 44 questions correctly. This can be cumbersome for patients, which compromises the reliability of their responses. Despite the fact that the accuracy of our model is not perfect (67%), it greatly reduces the number of required questions from 44 to 3–6. Furthermore, the development of our TCM BC online diagnosis system allows us to collect more data and increase the accuracy of our model for future work.

Coupled with the development of the TCM BCQ online diagnosis system, the BCQ is not only more practical and efficient but also establishes a system to facilitate future research.

## 5. Conclusions

In this study, we briefly explained the TCM BC types and described how BC is determined using the BCQ. We created a decision tree model for determining the TCM BC type using the BCQ dataset. Our approach achieved 67% accuracy for 4 single BC types. Using the decision tree model, we reduced the required number of the BCQ questions from 44 to 3–6. This allows our approach to considerably expedite the assessment process. In addition, we created a mobile phone app using this approach for practical and efficient usage. Using the app is more efficient in medical settings and helps improve the model by collecting more data.

### 5.1. Future Work

In subsequent studies, we plan to work with more doctors to use the BCQ app for collecting more BCQ data to create a more accurate BCQ decision tree model. Also, this paper only considers a single BC syndrome type. To improve reliability, mixed BC syndrome types can be analyzed after collecting a bigger dataset in the future. Additionally, the information obtained from analyzing a large amount of BCQ data can be used to revise the questions of the BCQ.

## Figures and Tables

**Table 1:** Question numbers for each BC type.

BC type	#Test
Yin-Xu	2, 4, 8, 10, 11, 16, 18, 20, 23, 26, 29, 30, 31, 32, 35, 37, 38, 39, and 40
Yang-Xu	3, 5, 8, 9, 15, 16, 17, 22, 23, 24, 28, 31, 33, 36, 37, 41, 42, 43, and 44
Phlegm and blood stasis	1, 4, 5, 6, 7, 12, 13, 14, 16, 17, 19, 20, 21, 25, 27, and 34

**Table 2:** Criteria for determining the BC type.

BC type	Number of questions	Score	Judgment criteria
Yin-Xu	19	1–5	30
Yang-Xu	19		31
Phlegm and blood stasis	16		27

## Data Availability

The data used to support the decision tree of this study are available from the corresponding author upon request.
